# Effects of 12 Weeks of Resistance Training on Body Composition, Muscle Hypertrophy and Function, Blood Lipid Level, and Hemorheological Properties in Middle-Aged Obese Women

**DOI:** 10.31083/j.rcm2407196

**Published:** 2023-07-12

**Authors:** Jisoo Seo, Hun-Young Park, Won-Sang Jung, Sung-Woo Kim, Yerin Sun, Jae-Ho Choi, Jisu Kim, Kiwon Lim

**Affiliations:** ^1^Department of Sports Medicine and Science, Konkuk University, 05029 Seoul, Republic of Korea; ^2^Physical Activity and Performance Institute (PAPI), Konkuk University, 05029 Seoul, Republic of Korea; ^3^Department of Senior Exercise Prescription, Dongseo University, 47011 Busan, Republic of Korea; ^4^Department of Physical Education, Konkuk University, 05029 Seoul, Republic of Korea

**Keywords:** resistance training, muscle strength, erythrocyte, hemorheology, RBC aggregation, RBC deformability, obesity

## Abstract

**Background:**

This study investigated the effects of 12-week resistance training on body composition, blood pressure, blood lipid levels, muscle cross-sectional area (CSA), isokinetic muscle function, and hemorheological properties in middle-aged obese women.

**Methods:**

Twenty-eight obese women with a mean age of 50.79 ± 5.80 years were randomly assigned to the control (CON, n = 13) or experimental (EXP, n = 15) group. The EXP group underwent a resistance training program composed of warm-up, main resistance exercise (deadlift, barbell squat, seated leg extension, and lying leg curl, bench press, preacher bench biceps curl, barbell rowing, and dumbbell shoulder press), and cool-down. The resistance exercise consisted of three sets of 8–10 repetitions (reps) performed with 70–80% of 1-rep maximum, and reps and sets were increased every 3 weeks. The training frequency was 80 min, 3 days per week for 12 weeks. The CON group maintained their daily lifestyle without training. All participants underwent measurements of body composition (weight, body mass index, lean body mass, fat mass, and % body fat), blood pressure (systolic blood pressure, diastolic blood pressure, mean arterial pressure, and pulse pressure), blood lipid levels (triglycerides, total cholesterol, high-density lipoprotein cholesterol, and low-density lipoprotein cholesterol), CSA of the muscles (quadriceps, hamstring, and total thigh muscle), isokinetic muscle function (peak torque [PT], relative PT, mean power, and total work [TW]), and hemorheological properties (erythrocyte deformability and aggregation) before and after 12 weeks of training.

**Results:**

The EXP group showed a significant improved muscle function, including PT (*p <* 0.001), relative PT (*p <* 0.001) in extension 60°/s, TW (*p <* 0.001) in extension 180°/s, and TW (*p *= 0.018) in flexion 180°/s. Regarding hemorheological properties, the EXP group showed significant improvement in erythrocyte aggregation (*p <* 0.001) and deformability (*p <* 0.001).

**Conclusions:**

The present study verified that our resistance training program resulted in greater muscle function, decreased fat mass, and improved hemorheological properties.

**Clinical Trial Registration:**

This study was registered with cris.nih.go.kr (No. KCT0007412).

## 1. Introduction

The World Health Organization (WHO) has defined obesity as “a condition in which there is an abnormal or excessive accumulation of fat that can harm health”; obesity is a major cause of harm to the health [1]. The Organisation for Economic Cooperation and Development (OECD)/WHO (2020) reported that in 2016, 39% of the men and 40% of the women (approximately 2 billion adults) aged 18 years and older worldwide were overweight, and 11% of the men and 15% of the women (more than 500 million adults) were obese. Both overweight status and obesity have significantly increased over the past 40 years (OECD/WHO, 2020). Globally, the prevalence of obesity has increased by approximately 50%, from 8.7% in 2000 to 13.1% in 2016. In 2016, the prevalence of obesity in men was 11.1%, whereas, in women, it was 15.1%, which is approximately 35% higher in women (WHO, 2016). A similar trend has been reported in Korea, with the obesity prevalence increasing by approximately 20%, from 29.7% in 2009 to 35.7% in 2018 [2].

As obesity is increasing worldwide, so are the risks of obesity-associated diseases and death, underscoring the importance of weight management strategies [3, 4, 5]. Diet control, exercise, drugs, and surgery have been the core of obesity management [6]. Exercise is an effective obesity treatment method because it can reduce body weight (BW) by maximizing health benefits [6, 7]. Exercise benefits include maintaining normal BW, reducing body mass index (BMI), reducing visceral fat, improving insulin sensitivity, improving blood sugar, lowering blood pressure, improving blood lipid levels, as well as improving musculoskeletal function, increasing lean body mass, and improving immune function [8, 9, 10].

Exercise for obesity treatment and health promotion can be divided into aerobic and resistance exercises. Aerobic exercise is a traditional method that can promote fat loss [11], and resistance exercise is valued as an additional approach for preserving lean and skeletal muscle mass [12]. As aerobic and resistance exercises have different effects on weight management, many previous studies have compared the effects of the two exercises [13, 14, 15]. Willis *et al*. [14] reported that aerobic exercise significantly reduced body fat more than resistance exercise, while resistance exercise significantly increased lean body mass. Yang *et al*. [15] conducted a meta-analysis of cardiovascular risk factors. Both aerobic and resistance exercises showed positive effects on increasing high-density lipoprotein cholesterol (HDL-C) and decreasing low-density lipoprotein cholesterol (LDL-C), total cholesterol (TC), and triglyceride (TG) levels, but no significant difference was found between the exercises. In a literature study published by Braith and Stewart [13], aerobic exercise was more effective in reducing fat mass, and resistance exercise was more effective in increasing lean body mass, but both exercises had similar positive effects on cardiovascular risk factors. In obese individuals, these findings showed that resistance exercise is relatively less effective in reducing body fat than aerobic exercise but is a useful exercise method that can improve lean body mass and cardiovascular risk factors.

The greatest benefit of resistance exercise is an increase in lean body mass [16]. It has been reported that people with low lean body mass have a high body-fat ratio, an increased risk of diabetes, and a high mortality rate [17]. Muscle mass generally peaks in the late 30s and thereafter declines by approximately 0.37% and 0.47% annually in women and men, respectively [18, 19, 20]. Low muscle mass is a predictor of cardiovascular mortality risk and significantly increases the risk of all-cause mortality [12, 21]. Therefore, obese people need to maintain appropriate muscle mass and optimal muscle strength levels through resistance exercise to reduce disease morbidity and mortality [22, 23].

Recently, hemorheological function, an index for blood circulation, component flow, and deformability, has received much attention as a health indicator related to obesity [24, 25]. Hemorheological properties are factors that regulate blood flow from microvessels to tissues, and the representative variables are red blood cell (RBC) deformability and aggregation [26, 27]. RBC deformability and aggregation are key determinants of vascular health, as RBCs must be deformed to pass through capillaries that are much narrower than RBCs to ensure sufficient blood flow in the microcirculation [26]. Obese people have lower RBC deformability and higher RBC aggregation than healthy people [28], and those with obesity-related diseases, such as cardiovascular disease and type 2 diabetes, show reduced RBC deformability and increased RBC aggregation [29, 30, 31]. Exercise has been reported as a representative method for improving hemorheological properties [32, 33]. In particular, aerobic exercise has been reported to improve RBC deformability and aggregation and to induce long-term improvement of the coronary microvascular system and vascular function, including resting blood pressure reduction and increase in peripheral blood flow [34, 35, 36, 37]. However, the effect of resistance exercise on hemorheological properties remains unclear [38]. Although previous studies have reported improvements in RBC deformability and aggregation according to acute resistance exercise [34], very few studies have examined the hemorheological properties according to long-term resistance training. The prevalence of obesity is increasing worldwide, and it is increasing more in women than in men. Women have less muscle mass than men, and especially in the case of middle-aged women, the risk of obesity after menopause is much greater. Thus, obese women were selected as participants. Therefore, this study examined the effects of 12 weeks of resistance training on body composition, muscle hypertrophy and function, blood lipid levels, and hemorheological properties in middle-aged obese women to assess the effectiveness of resistance exercise as an exercise intervention for obesity treatment. Resistance training for 12 weeks may positively affect body composition, muscle hypertrophy and function, blood lipid levels, and hemorheological properties in middle-aged obese women with a 25–30 ${\mathrm{kg/m}}^{2}$ BMI.

## 2. Methods

### 2.1 Participants

**Fig. 1.**
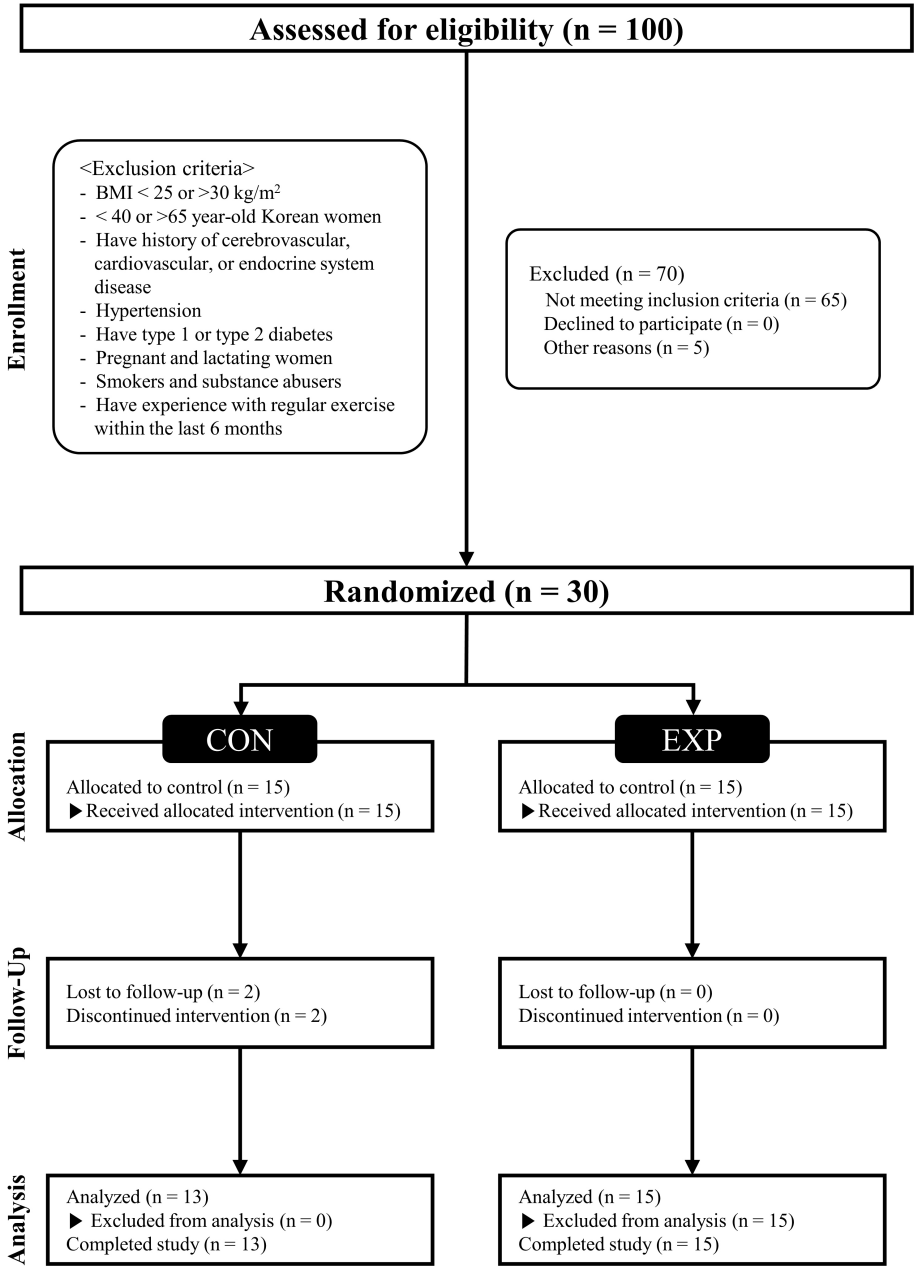
**CONSORT (Consolidated Standards of Reporting Trials) flow diagram**. CON, control group; EXP, experimental group; BMI, body mass index.

The study enrolled 28 obese middle-aged women with a mean age of 50.79 ± 5.80 years; participants’ physical characteristics are shown in Table 1. The inclusion criteria were as follows: (1) adult female aged 40–65 years; (2) with a BMI of 25–30 ${\mathrm{kg/m}}^{2}$; (3) had not exercised in the last 6 months; (4) willing to fully participate in the exercise program; and (5) consent to participate in this study and voluntarily signed the written consent form. The exclusion criteria were as follows: (1) severe cerebrovascular, cardiovascular, or endocrine system disease over the 6 months prior to the study; (2) uncontrolled hypertension; (3) type 1 or type 2 diabetes; (4) pregnant and lactating women; and (5) smokers and substance abusers (including alcohol). Those who met the inclusion criteria were randomly assigned a control group (CON, n = 15) that led their daily lives and an experimental group (EXP, n = 15) that underwent resistance training; however, two participants in the CON voluntarily withdrew for personal reasons, and 13 participants in the CON finally participated. The EXP underwent resistance training 3 days per week for 12 weeks, and the CON maintained their normal daily life for 12 weeks. After a sufficient explanation of the experiment and possible adverse effects, the participants signed the informed consent form before the start of the study. The consolidated standards of reporting trial (CONSORT: Consolidated Standards of Reporting Trials) flow diagram is shown in Fig. 1. This study was approved by the Institutional Review Board of Konkuk University (7001355-202201-E-161) in Korea. All study procedures were performed in accordance with the Helsinki Declaration.

**Table 1.** **Participants’ characteristics**.

Variables	CON (n = 13)	EXP (n = 15)	*p-*value
Height (cm)	158.08 ± 5.97	159.22 ± 4.65	0.359
Body weight (kg)	72.73 ± 10.27	69.85 ± 7.76	0.565
BMI (${\mathrm{kg/m}}^{2}$)	29.12 ± 4.01	27.90 ± 2.03	0.105
Lean body mass (kg)	39.95 ± 3.33	39.01 ± 4.31	0.375
Fat mass (kg)	31.28 ± 7.70	28.89 ± 4.33	0.248
Percent body fat (%)	42.12 ± 5.39	41.23 ± 2.94	0.238

Note: Data are means (± SD). SD, standard deviation; CON, control group; EXP, experimental group; BMI, body mass index.

### 2.2 Study Design

**Fig. 2.**
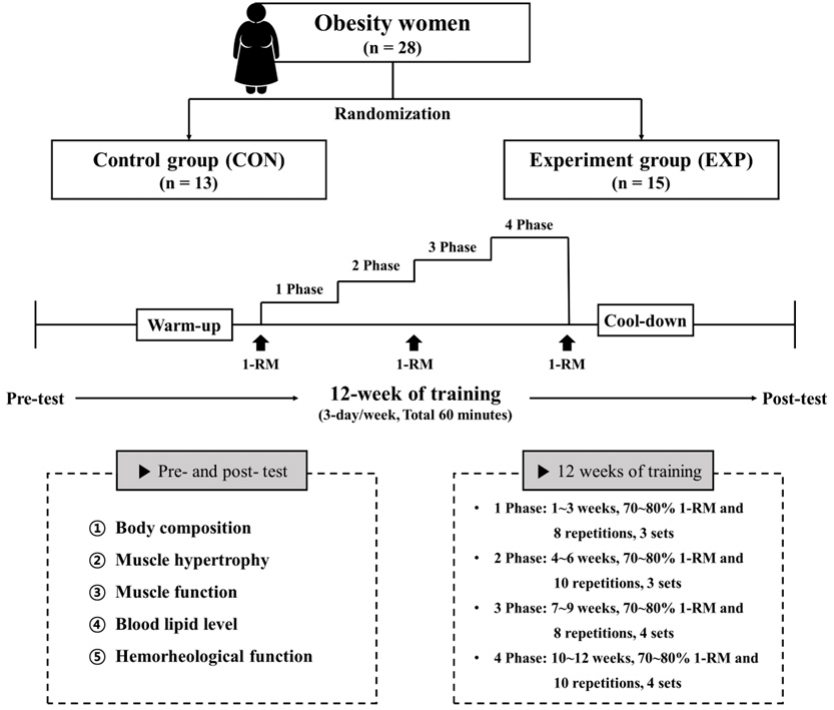
**Study design**. 1-RM, one repetition maximum.

The study design involved a 1-day pre-testing, a 12-week intervention, and a 1-day post-testing. The study design is illustrated in Fig. 2.

On the pre- and posttesting days, all participants fasted for more than 8 h, and after stabilization, their blood pressure and body composition were measured between 7:00 and 9:00 AM. Blood samples were collected from the fingertips (20 µL), and venous (10 mL) by a trained nurse in a sitting position. The skinfold and circumference of the waist, hip, triceps, biceps, and thigh were measured to calculate the cross-sectional area (CSA) [39]. Isokinetic muscle function was measured at 60° for muscle strength (peak torque [PT], PT/BW) and 180° for muscular endurance (mean power, total work [TW]).

After the testing session, the participants were randomly assigned to the CON, which maintained the same daily lifestyle as before, or the EXP, which underwent a resistance training program. The resistance training program consisted of 10 min of warm-up, 60 min of the main exercise, and 10 min of cool-down; the rest period between each exercise and the next was set within 1–2 min. The warm-up and cool-down consisted of dynamic and static stretching, respectively; resistance training consisted of four movements of the upper body (bench press, preacher bench biceps curl, barbell rowing, and dumbbell shoulder press) and four movements of the lower body (deadlift, barbell squat, seated leg extension, and lying leg curl). The main resistance exercise consisted of 3–4 sets of 8–10 repetitions at 70–80% of one repetition maximum (1-RM) after measuring 1-RM. The training frequency was 80 min, 3 days per week, for 12 weeks. The number of repetitions and sets was increased every 3 weeks according to the principle of increasing the load of exercise prescription (Fig. 2). The resistance training program were performed at a gym in Seoul, and a health trainer residing at the gym fully understood our training program and guided the participants to perform the exercise.

### 2.3 Measurement

#### 2.3.1 Body Composition

Body composition parameters, including BW (kg), lean body mass (kg), fat mass (kg), and body fat percentage (%), of all participants were estimated using a bioelectrical impedance analyzer (Inbody 770, Inbody, Seoul, Korea), and BMI (${\mathrm{kg/m}}^{2}$) was calculated using formulae for height and weight. All participants wore lightweight clothing and were asked to remove all metallic items from their bodies.

#### 2.3.2 Blood Pressure

The resting blood pressure, systolic blood pressure (SBP), and diastolic blood pressure (DBP) were measured twice in the sitting position after a minimum of 5 min rest using an automatic sphygmomanometer (HBP-9020, OMRON Colin, Tokyo, Japan). The average value was used for analyses and calculation of mean arterial blood pressure (mean arterial blood pressure (MAP) = DBP + [SBP – DBP] / 3) and pulse pressure (pulse pressure (PP) = SBP – DBP).

#### 2.3.3 Muscle Hypertrophy

To evaluate muscular hypertrophy, the CSA was calculated by measuring the skinfold and circumference of the waist, hip, triceps, biceps, and thigh [40, 41]. The waist and hip circumferences were measured twice with a tapeline, with the participant standing on a flat floor, the average was used, and the unit was measured to 0.1 cm. The thigh circumference was measured twice using a tapeline with the participant standing in an upright position with both legs 10–15 cm apart and the weight of both feet equal; the average was used, and the unit was measured to 0.1 cm. The skinfold of the thigh was measured by grabbing fat from the front of the thigh between the hip and knee using the thumb and index finger, measured twice, and the average was used, and the unit was measured to 0.1 cm.

The quadriceps, hamstring, and total thigh muscle CSAs were calculated using the following formula [39]: quadriceps CSA (${\mathrm{cm}}^{2}$) = (2.52 × thigh circumference) – (1.25 × thigh skin folder) – 45.13, hamstring CSA (${\mathrm{cm}}^{2}$) = (1.08 × thigh circumference) – (0.64 × thigh skin folder) – 22.69, and total thigh muscle CSA (${\mathrm{cm}}^{2}$) = (1.08 × thigh circumference) – (2.09 × thigh skin folder) – 80.99.

#### 2.3.4 Muscle Function

Muscle function was measured using Biodex (Biodex Medical Systems, New York, NY, U.S.). With the participant sitting on the measuring chair, the center point of the joint was adjusted using a table and backrest to coincide with the dynamometer’s axis of rotation. During flexion and extension exercises, the thigh and chest were fixed to prevent external force application to other body parts than the exercised part. The range of motion of the knee joint was designated as 0° of extension and 90° of flexion and was measured after performing gravity correction to exclude the effect of gravity. Through this, isokinetic muscle strength and muscular endurance were measured. For muscle strength measurement, the PT and relative PT (PT/BW) were calculated by performing extension and flexion with a maximum force four times at an angular velocity of 60°/s. Muscle endurance was measured 11 times at an angular velocity of 180°/s to calculate the average power and TW.

#### 2.3.5 Blood Lipid Level

Blood lipid levels were measured using Lipidocare (SD Biosensor, Inc., Seoul, Korea). After fasting for more than 8 h the day before the test, blood samples were collected, and lipid levels, including TG, TC, HDL-C, and LDL-C, were measured using the fingertips method.

#### 2.3.6 Hemorheological Properties

RBC deformability and aggregation were evaluated as hemorheological parameters to evaluate microvascular circulation function. RBC deformability and aggregation were analyzed using Rheoscan-D (Rheo Meditech Inc., Seoul, Korea) under environmental conditions of 25 °C and 3 Pa shear stress within 4–6 h after blood collection. RBC deformability was measured using the elongation index (EI) by first transferring the sample into a 2 mL microseparation tube, which was diluted in 700 µL of 5.5% polyvinylpyrrolidone (360 kDa) dissolved in 1 mmol phosphate-buffered saline (pH 7.4; osmolality: 300 mOsmol/kg) in a K3-ethylenediaminetetraacetic acid tube (Greiner Bio-one, Chon Nuri, Thailand). This solution (0.5 mL) was then analyzed using a D-test kit, according to the manufacturer’s instructions. The accuracy of the RBC EI was measured using a Lineweaver–Burk plot model. RBC aggregation was measured using the aggregation index (AI), and 8 µL of the whole blood sample was analyzed using an A-test kit, according to the manufacturer’s instructions.

### 2.4 Statistical Analysis

With the aid of the G*power analysis tool, the sample size was determined. Based on data from a similar study in healthy women, a power analysis using an ɑ-level and power of 0.05 and 0.90, respectively, showed a sufficient samplesize of 18 participants. All statistical analyses were conducted using the SPSS software (version 25.0; IBM Corp., Armonk, NY, USA) for Windows. Data were presented as mean ± standard deviation. The normality assumption was confirmed using the Shapiro–Wilks test before performing parametric statistics. A two-way analysis (time × group) of variance with repeated measures of the “time” factor was used to analyze the effects of the training programs on each dependent variable. A paired *t*-test was used to compare the post-training and pre-training values of the dependent variables in each group separately. In addition, the Cohen’s d, an effect size that reflects statistical values calculated from data samples and standardized mean differences, was calculated for clinical interpretation of the data. The statistical difference in the means was determined using the significance level and 95% confidence interval (CI). Significant effects were evaluated using the Cohen’s d effect size (small d = 0.2, medium d = 0.5, and large d = 0.8 effect size). The level of significance was set a priori at *p* < 0.05.

## 3. Results

### 3.1 Body Composition

As shown in Table 2, there were no significant interactions with any body composition parameters. A significant main effect of the time was observed on body fat. However, there were no changes in BW, BMI, lean body mass, or body fat percentage.

**Table 2.** **Pre- and post-intervention data (means ± SD) for body composition with main analysis of variance results**.

Variables	CON (n = 13)			EXP (n = 15)			*p* ($\eta^{2}$) value		
	Pre	Post	Cohen’s d (95% CI)	Pre	Post	Cohen’s d (95% CI)	T	G	Inter
Body weight (kg)	72.7 ± 10.3	71.9 ± 9.6	–0.08 (–0.93, 0.77)	69.9 ± 7.8	69.3 ± 8.4	–0.06 (–0.91, 0.79)	0.104 (0.098)	0.426 (0.025)	0.713 (0.005)
BMI (${\mathrm{kg/m}}^{2}$)	29.1 ± 4.0	28.8 ± 3.8	–0.08 (–0.92, 0.78)	27.5 ± 2.0	27.3 ± 2.4	–0.07 (–0.92, 0.79)	0.117 (0.092)	0.192 (0.065)	0.768 (0.003)
Lean body mass (kg)	40.0 ± 3.3	39.4 ± 3.8	–0.15 (–1.00, 0.70)	39.0 ± 4.3	39.1 ± 4.0	0.03 (–0.82, 0.88)	0.316 (0.039)	0.693 (0.006)	0.131 (0.086)
Fat mass (kg)	31.3 ± 7.7	30.5 ± 7.2	–0.10 (–0.95, 0.75)	28.9 ± 4.3	28.2 ± 5.0	–0.14 (–0.99, 0.72)	${\mathrm{0.017}}^{†}$(0.201)	0.312 (0.039)	0.932 (0.000)
Percent body fat (%)	42.1 ± 5.4	41.9 ± 5.2	–0.03 (–0.88, 0.82)	41.2 ± 2.9	40.4 ± 3.2	–0.25 (–1.10, 0.61)	0.101 (0.100)	0.458 (0.021)	0.289 (0.043)

Note: SD, standard deviation; CI, confidence interval; CON, control group; EXP, experimental group; T, time; G, group; Inter, interaction. ^†^Significant interaction or main effect; BMI, body mass index.

### 3.2 Blood Pressure

There were no significant interactions or main effects in all blood pressure parameters, and there were no significant differences according to resistance exercise for 12 weeks, as shown in Table 3.

**Table 3.** **Pre- and post-intervention data data (means ± SD) for blood pressure with main analysis of variance results**.

Variables	CON (n = 13)			EXP (n = 15)			*p* ($\eta^{2}$) value		
	Pre	Post	Cohen’s d (95% CI)	Pre	Post	Cohen’s d (95% CI)	T	G	Inter
SBP (mmHg)	127.5 ± 16.3	126.2 ± 18.4	–0.07 (–0.92, 0.78)	125.1 ± 10.1	124.8 ± 17.9	–0.01 (–0.86, 0.85)	0.771 (0.003)	0.732 (0.005)	0.847 (0.001)
DBP (mmHg)	77.3 ± 10.6	75.9 ± 9.4	–0.14 (–0.98, 0.72)	78.9 ± 7.3	76.0 ± 11.8	–0.19 (–1.04, 0.67)	0.261 (0.048)	0.792 (0.003)	0.687 (0.006)
MAP (mmHg)	94.0 ± 12.0	92.7 ± 12.3	–0.11 (–0.96, 0.74)	94.3 ± 7.9	92.3 ± 13.5	–0.06 (–0.91, 0.79)	0.425 (0.025)	0.988 (0.000)	0.873 (0.001)
PP (mmHg)	50.2 ± 9.4	50.2 ± 9.9	0.01 (–0.84, 0.86)	46.1 ± 5.6	48.8 ± 8.5	0.35 (–0.52, 1.20)	0.312 (0.039)	0.353 (0.033)	0.340 (0.035)

Note: SD, standard deviation; CI, confidence interval; CON, control group; EXP, experimental group; T, time; G, group; Inter, interaction; SBP, systolic blood pressure; DBP, diastolic blood pressure; MAP, mean arterial pressure; PP, pulse pressure.

### 3.3 Muscle Hypertrophy

As shown in Table 4, there were no significant interactions or main effects in muscle hypertrophy, and there was no significant difference after the resistance exercise for 12 weeks.

**Table 4.** **Pre- and post-intervention data (means ± SD) for muscle hypertrophy with main analysis of variance results**.

Variables	CON (n = 13)			EXP (n = 15)			*p* ($\eta^{2}$) value		
	Pre	Post	Cohen’s d (95% CI)	Pre	Post	Cohen’s d (95% CI)	T	G	Inter
Quadriceps CSA (${\mathrm{cm}}^{2}$)	44.97 ± 6.14	42.84 ± 7.03	–0.32 (–1.17, 0.54)	47.05 ± 9.21	47.18 ± 8.17	0.01 (–0.84, 0.87)	0.376 (0.030)	0.252 (0.050)	0.318 (0.038)
Hamstrings CSA (${\mathrm{cm}}^{2}$)	12.31 ± 2.40	11.34 ± 2.87	–0.36 (–1.21, 0.51)	13.53 ± 3.70	13.45 ± 3.37	–0.02 (–0.87, 0.83)	0.306 (0.040)	0.137 (0.083)	0.381 (0.030)
Total thigh muscle CSA (${\mathrm{cm}}^{2}$)	94.36 ± 12.10	90.54 ± 13.58	–0.29 (–1.14, 0.57)	97.49 ± 17.77	98.03 ± 15.57	0.03 (–0.82, 0.88)	0.421 (0.025)	0.329 (0.037)	0.288 (0.043)

Note: SD, standard deviation; CI, confidence interval; CON, control group; EXP, experimental group; T, time; G, group; Inter, interaction; CSA, cross-sectional areas.

### 3.4 Muscle Function

As shown in Table 5, a significant interaction was observed in PT and PT/BW of 60°/s extension, TW of 180°/s extension, and a significant main effect of the time was found in TW of 180°/s flexion. The post-hoc analysis revealed a tendency to decrease PT of 60°/s extension in the CON (*p* = 0.064), but the EXP increased significantly with significantly improved PT/BW of 60°/s extension, TW of 180°/s extension.

**Table 5.** **Pre- and post-intervention data (means ± SD) for muscle function with main analysis of variance results**.

Variables		CON (n = 13)			EXP (n = 15)			*p* (*$\eta^{2}$*) value		
		Pre	Post	Cohen’s d (95% CI)	Pre	Post	Cohen’s d (95% CI)	T	G	Inter
Ex	60°/sec PT (N⋅m)	109.90 ± 24.98	104.12 ± 23.99	–0.24 (–1.08, 0.62)	110.19 ± 25.01	118.72 ± 23.11	0.33^***^ (–0.54, 1.18)	0.364 (0.032)	0.419 (0.025)	${\mathrm{0.000}}^{†}$ (0.471)
	60°/sec PT/BW (%)	151.05 ± 22.21	144.93 ± 20.66	–0.28 (–1.13, 0.58)	156.71 ± 24.68	168.98 ± 20.89	0.49^***^ (–0.39, 1.35)	0.118 (0.091)	0.082 (0.112)	${\mathrm{0.000}}^{†}$ (0.473)
	180°/sec MP (W)	85.00 ± 19.60	85.05 ± 21.06	0.00 (–0.85, 0.85)	94.05 ± 24.78	100.38 ± 26.38	0.23 (–0.63, 1.08)	0.060 (0.130)	0.172 (0.070)	0.064 (0.126)
	180°/sec TW (J)	784.32 ± 157.00	785.42 ± 171.53	0.01 (–0.84, 0.85)	866.75 ± 212.88	937.85 ± 237.05	0.31^***^ (–0.55, 1.16)	${\mathrm{0.024}}^{†}$ (0.182)	0.125 (0.088)	${\mathrm{0.028}}^{†}$ (0.173)
Fl	60°/sec PT (NM)	50.25 ± 14.05	50.05 ± 16.32	–0.01 (–0.86, 0.84)	51.11 ± 10.25	54.13 ± 9.62	0.29 (–0.58, 1.14)	0.269 (0.047)	0.598 (0.011)	0.208 (0.060)
	60°/sec PT/BW (%)	68.82 ± 12.31	69.15 ± 13.27	0.03 (–0.82, 0.87)	72.74 ± 10.80	77.29 ± 10.28	0.43 (–0.45, 1.28)	0.154 (0.076)	0.152 (0.077)	0.216 (0.058)
	180°/sec MP (W)	42.05 ± 15.81	42.53 ± 16.32	0.03 (–0.82, 0.88)	46.13 ± 7.31	50.41 ± 7.96	0.51 (–0.37, 1.36)	0.106 (0.097)	0.188 (0.066)	0.192 (0.064)
	180°/sec TW (J)	430.16 ± 125.56	446.20 ± 156.29	0.11 (–0.74, 0.96)	471.76 ± 77.74	518.73 ± 83.24	0.58 (–0.30, 1.44)	${\mathrm{0.047}}^{†}$ (0.144)	0.166 (0.072)	0.314 (0.039)

Note: SD, standard deviation; CI, confidence interval; CON, control group; EXP, experimental group; T, time; G, group; Inter, interaction; Ex, extension; Fl, flexion; PT, peak torque; BW, body weight; MP, mean power; TW, total work. ^†^Significant interaction or main effect. ^***^p < 0.001 vs. before intervention.

### 3.5 Blood Lipid Level

As shown in Table 6, there were no significant interactions or main effects on blood lipid levels, and there was no significant difference according to resistance exercise for 12 weeks.

**Table 6.** **Pre- and post-intervention data (means ± SD) for blood lipid level with main analysis of variance results**.

Variables	CON (n = 13)			EXP (n = 15)			*p* (*$\eta^{2}$*) value		
	Pre	Post	Cohen’s d (95% CI)	Pre	Post	Cohen’s d (95% CI)	T	G	Inter
TG (mg/dL)	151.5 ± 102.6	137.7 ± 70.2	–0.15 (–1.00, 0.70)	107.1 ± 59.2	126.6 ± 85.1	0.12 (–0.74, 0.97)	0.840 (0.002)	0.314 (0.039)	0.243 (0.052)
TC (mg/dL)	209.6 ± 61.2	199.5 ± 44.8	–0.17 (–1.02, 0.68)	194.5 ± 49.7	184.3 ± 41.1	–0.20 (–1.05, 0.66)	0.076 (0.116)	0.406 (0.027)	0.992 (0.000)
HDL-C (mg/dL)	55.7 ± 11.1	53.5 ± 12.5	–0.19 (–1.03, 0.67)	56.6 ± 12.8	53.5 ± 14.8	–0.17 (–1.02, 0.68)	0.118 (0.091)	0.917 (0.000)	0.801 (0.002)
LDL-C (mg/dL)	112.2 ± 30.0	112.1 ± 30.4	0.00 (–0.85, 0.85)	118.7 ± 36.6	107.6 ± 31.4	–0.32 (–1.17, 0.55)	0.194 (0.064)	0.930 (0.000)	0.200 (0.062)

Note: SD, standard deviation; CI, confidence interval; CON, control group; EXP, experimental group; T, time; G, group; Inter, interaction; TG, triglyceride; TC, total cholesterol; HDL-C, high-density lipoprotein cholesterol; LDL-C, low-density lipoprotein cholesterol.

### 3.6 Hemorheological Properties

**Fig. 3.**
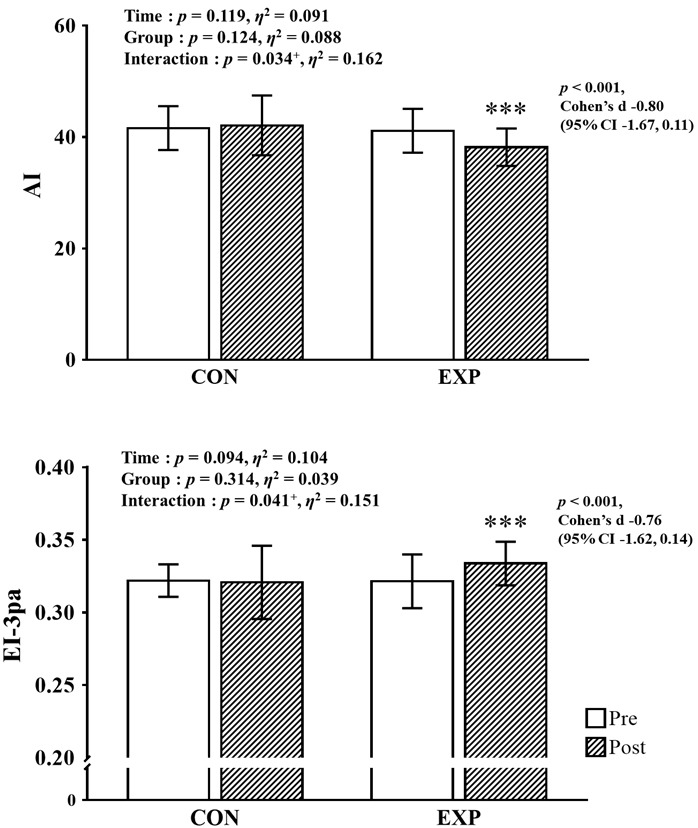
**Pre- and post-intervention data for hemorheological function with main analysis of variance results**. CI, confidence interval; CON, control group; EXP, experimental group; AI, aggregation index; EI, elongation index. ^+^Significant interaction or main effect; ^***^p < 0.001 vs. before intervention.

As shown in Fig. 3, a significant interaction was observed both the AI (*p* < 0.05, $\eta^{2}$ = 0.162) and EI-3pa (*p* < 0.05, $\eta^{2}$ = 0.151). The post-hoc analysis revealed a significant decrease in AI (Cohen’s d = –0.80, 95% CI –1.67, 0.10) and a significant increase in EI-3pa (Cohen’s d = –0.76, 95% CI –1.62, 0.14) in the EXP.

## 4. Discussion

This study aimed to confirm the effects of 12 weeks of resistance exercise on body composition, muscle hypertrophy and function, blood lipid levels, and hemorheological properties in middle-aged obese women. In line with the study hypothesis, muscle function, and hemorheological properties significantly improved in the EXP after the resistance exercise.

### 4.1 Body Composition

After 12 weeks of resistance training, no significant changes in body composition were observed for both CON and EXP. As for the improvement of body composition following the resistance exercise program, studies have reported relatively diverse results according to the duration, intensity, frequency, and participants’ characteristics of the resistance exercise program, but most of these studies demonstrated the program’s effectiveness in maintaining lean body mass and reducing body fat percentage [42]. Contrary to the results of previous studies, this study showed no significant improvement in lean body mass was observed in the EXP following the resistance exercise program. Unlike previous studies, in which the resistance exercise program was applied to many participants for 6–8 months, this study applied a 3-month resistance exercise program to relatively few participants, possibly accounting for the no significant improvement in lean body mass [14, 43]. In addition, failure to conduct dietary intake education and research, which is important for lean body mass formation, during the study period may have also accounted for the no significant improvement in lean body mass. However, the CON tended to show decreased lean body mass in this study. In contrast, the EXP showed a relatively increased tendency, thought to be because the resistance exercise program suppressed the aging-associated decrease in lean body mass. Therefore, as in previous studies, a long-term resistance exercise program application could significantly improve body composition in the EXP.

### 4.2 Blood Pressure

Several systematic reviews and meta-analyses of previous studies on the relationship between resistance exercise programs and blood pressure have reported that long-term resistance exercise programs improve blood pressure and cardiovascular disease risk factors in various participants (e.g., obesity, metabolic syndrome, and cardiovascular disease) [44, 45, 46]. Following the examination of the 12-week resistance exercise program effect on blood pressure in this study, there was no significant change in SBP, DBP, MAP, and PP in the CON and EXP; these results were consistent with those of Tibana *et al*. [47] and Cortez-Cooper *et al*. [48]. The absence of change in blood pressure may be due to the relatively short training period (12 weeks), similar to previous studies with the same results, and all participants’ initial blood pressure were normal or elevated levels. In addition, this is supported by the American College of Sports Medicine (ACSM) report that the decrease in blood pressure following long-term exercise treatment is related to the initial blood pressure level (ACSM, 2004).

### 4.3 Muscle Hypertrophy and Function

Several previous studies reported that resistance exercise programs significantly increased CSA [49, 50], and that high-intensity exercise was more effective for muscle hypertrophy than low-intensity exercise [51, 52]. However, in this study, despite the intervention of a high-intensity resistance exercise program of similar duration and intensity to previous studies that showed significant improvement in CSA, no significant changes were observed in the quadriceps, hamstrings, and total thigh muscle CSA. Although previous studies reported that differences in macronutrient intake, especially protein intake, could affect the muscle hypertrophy effect of resistance exercise programs [53, 54], this study did not control participants’ diet and did not conduct nutrition education and surveys. Therefore, it can be inferred that sufficient protein intake for muscle synthesis was not achieved [55]. In addition to protein intake, testosterone, an androgen anabolic hormone, also affects muscle hypertrophy. Generally, testosterone levels rise immediately after a high-intensity resistance exercise program in men. However, the results of studies on testosterone response to a resistance exercise program in women are uncertain. In addition, testosterone levels decrease gradually in women until menopause and then decrease rapidly after menopause [56]. Considering muscle protein synthesis response in older people is reported to be lower than that in younger individuals [57], the relatively short program duration is believed to be insufficient for muscle hypertrophy to occur in “middle-aged women” in this study [58]. In this study, although not significant, CSA tended to decrease in the CON and increased or maintained in the EXP following the 12-week resistance exercise program; if a long-term resistance exercise program of more than 6 months is applied in further studies, CSA may improve as in previous studies [50].

The increase in muscle CSA and development of muscle function are highly correlated [59], but the development of muscle function also occurs through a combination of muscle morphological improvement and neurological development [60]. The development of muscle function following the resistance exercise program could be mediated by increased muscle hypertrophy or activation of the motor unit, a unit of nerves that controls muscles [61, 62]. Milner-Brown and Lee [63] reported that a 6-week resistance exercise program improved muscle function by increasing motor unit synchronization. Aagaard *et al*. [64] suggested that a high-intensity resistance exercise program could activate motor units, thereby improving muscle function. In addition, Toth *et al*. [65] and Brandenburg and Docherty [66] reported that muscle strength increased significantly through a resistance exercise program, although muscle hypertrophy, such as increased CSA, did not occur. These results prove that muscle function was significantly increased by the resistance exercise program in our study, despite the absence of muscle hypertrophy, such as increased CSA. In addition, as the training volume increases, the ratio of type Ⅱx, which has a relatively large CSA area and high conduction speed, decreases, and the ratio of Ⅱx can be maintained as the volume is moderate or low [67]. It is believed that the development of muscle function without CSA increase in the participants in this study was due to the moderate or low training volume. However, the inability to measure and interpret the results of muscle activation was a limitation of this study.

### 4.4 Blood Lipid Level

In previous studies that conducted systematic analysis and meta-analysis on blood lipid levels improvement through resistance exercise programs, it was reported that long-term resistance exercise programs effectively improved blood lipid levels [44, 45, 46]. According to the guidelines for exercise and physical activity of ACSM, resistance exercise can increase HDL-C and decrease LDL-C and TG levels (ACSM, 2009). Some previous studies reported significant improvements in participants’ TG, TC, HDL-C, and LDL-C levels following resistance exercise programs [68, 69], but Olson *et al*. [70] and Banz *et al*. [71] reported no significant improvement in blood lipid levels after a resistance exercise program.

Consistent with the findings of Olson *et al*. [70], this study did not show any change in blood lipid levels after the 12-week resistance exercise program. Although the participants in this study were obese middle-aged women, their blood lipid levels were within a relatively normal range before exercise; thus, no improvement in blood lipid levels was reported following the resistance exercise program. In the absence of a change in diet, improving body composition and metabolic profile is associated with increased lean body mass and increased type Ⅰ and Ⅱa CSA [72]. No increase in lean body mass or CSA was observed in this study, suggesting that a healthier metabolic profile was not observed. In addition, although blood lipid levels are greatly influenced by diet, there are limitations in interpreting the results of this study because nutrition education and surveys were not performed on the participants.

### 4.5 Hemorheological Properties

Hemorheological properties refer to the physical properties of the blood cells that play an important role in circulation to tissues through microvessels. The most representative variables include blood viscosity, plasma coagulation protein, RBC deformability, and aggregation [73, 74]. The major function of RBCs is to facilitate the exchange of oxygen and carbon dioxide with the surrounding tissues in the microcirculation. In practice, RBCs sometimes need to be deformed to flow through capillaries smaller than their size [75]. An increase in blood viscosity increases the frictional force on the blood vessel wall, thereby reducing blood flow, which is explained by the term shear rate and acts as a limiting factor in the oxygen-carrying capacity, increasing the induction rate of heart disease due to obesity. Conversely, reduced blood and plasma viscosity increase the arteriovenous oxygen difference, positively affecting obesity and lifestyle diseases [33, 74, 76, 77]. Regular exercise increases vasodilation, upregulates endothelial nitric oxide (NO) synthase to increase blood flow, as well as stimulates bone marrow activity, and upregulates factors that increase RBC production. However, studies on improving hemorheological properties through exercise have mainly focused on aerobic exercise. Only a few studies have confirmed the improvement in RBC deformability and aggregation after resistance exercise. Therefore, this study aimed to examine the effect of a 12-week resistance exercise program on the hemorheological properties of middle-aged obese women.

Cakir-Atabek *et al*. [34] divided young male participants into two groups: moderate-intensity (70% 1-RM, three sets of 12 repetitions) and high-intensity (85% 1-RM, three sets of 6 repetitions), and investigated the effect of a 6-week resistance exercise program on RBC deformation and aggregation. They reported a significant improvement in RBC deformability in both groups, but the moderate-intensity resistance exercise program showed a greater improvement in RBC deformability than the high-intensity exercise program. Although the participant characteristics and exercise types are different, Kim *et al*. [78] examined the effect of a 12-week complex exercise program consisting of resistance and aerobic exercise on hemorheological properties in obese older men; the long-term complex exercise program was effective in improving RBC deformability and aggregation. Simmonds *et al*. [79] reported that when an older person with a history of type 2 diabetes performed aerobic exercise for 12 weeks, the RBC AI significantly decreased, and the EI increased in older women. Through a literature study, Hu and Lin [80] found that the ratio of young RBCs with excellent deformability was higher in people who continuously exercised. It has been reported that this is because many RBCs with weakened deformability due to the end of their lifespan of 90–120 days are removed when exercise is performed. In addition, Smith *et al*. [81] argued that the improvement in hemorheological properties through exercise is due to the upregulation of erythropoietin, a hormone that regulates the production of RBCs.

Similar to previous studies, this study’s 12-week resistance exercise program significantly improved the RBC EI and AI in middle-aged obese women. This is thought to be caused by increased blood demand for muscle tissues, such as vascular elasticity and vascular endothelial cell function enhancement by NO, which are adaptive phenomena that occur through resistance exercise [82, 83]. Further studies are necessary to understand the effect of the resistance exercise program on hemorheological properties and more details of the mechanisms.

### 4.6 Limitations

This study had several limitations. First, although the participants of this study were middle-aged women aged 50.79 ± 5.80 years, menstrual cycle and sex hormones were not measured, and some participants were possibly postmenopausal women. Second, participants’ daily physical activities were not investigated. The results may vary depending on the participants’ physical abilities. Third, this study did not control the participants’ diet during the exercise intervention period and did not conduct nutrition education and surveys.

This should explore the significance of the results of the work, not repeat them. A combined Results and Discussion section is often appropriate. Avoid extensive citations and discussion of published literature.

## 5. Conclusions

This study confirmed that a 12-week resistance exercise program effectively maintained lean body mass, improved muscle function, and improved RBC aggregation and deformability in middle-aged obese women. However, studies on the effects of resistance training on hemorheological properties are lacking. Further studies are needed to investigate the effects of resistance exercise programs on hemorheological properties according to various loads, intensities, frequencies, times, and durations.

## Data Availability

The datasets used and/or analyzed during the current study are available from the corresponding author on reasonable request.
